# Retigeric Acid B Exhibits Antitumor Activity through Suppression of Nuclear Factor-κB Signaling in Prostate Cancer Cells *in Vitro* and *in Vivo*

**DOI:** 10.1371/journal.pone.0038000

**Published:** 2012-05-29

**Authors:** Yong-Qing Liu, Xiao-Yan Hu, Tao Lu, Yan-Na Cheng, Charles Y. F. Young, Hui-Qing Yuan, Hong-Xiang Lou

**Affiliations:** 1 Department of Biochemistry and Molecular Biology, Shandong University School of Medicine, Jinan, China; 2 Department of Natural Product Chemistry, Shandong University School of Pharmaceutical Sciences, Jinan, China; 3 Department of Pharmacology and Toxicology, Department of Biochemistry and Molecular Biology, Indiana University School of Medicine, Indianapolis, Indiana, United States of America; 4 Department of Pharmacology, Shandong University School of Pharmaceutical Sciences, Jinan, China; 5 Department of Urology, Mayo Clinical College of Medicine, Mayo Clinic, Rochester, Minnesota, United States of America; Wayne State University School of Medicine, United States of America

## Abstract

Previously, we reported that retigeric acid B (RB), a natural pentacyclic triterpenic acid isolated from lichen, inhibited cell growth and induced apoptosis in androgen-independent prostate cancer (PCa) cells. However, the mechanism of action of RB remains unclear. In this study, we found that using PC3 and DU145 cells as models, RB inhibited phosphorylation levels of IκBα and p65 subunit of NF-κB in a time- and dosage-dependent manner. Detailed study revealed that RB blocked the nuclear translocation of p65 and its DNA binding activity, which correlated with suppression of NF-κB-regulated proteins including Bcl-2, Bcl-x_L_, cyclin D1 and survivin. NF-κB reporter assay suggested that RB was able to inhibit both constitutive activated-NF-κB and LPS (lipopolysaccharide)-induced activation of NF-κB. Overexpression of RelA/p65 rescued RB-induced cell death, while knockdown of RelA/p65 significantly promoted RB-mediated inhibitory effect on cell proliferation, suggesting the crucial involvement of NF-κB pathway in this event. We further analyzed antitumor activity of RB in *in vivo* study. In C57BL/6 mice carrying RM-1 homografts, RB inhibited tumor growth and triggered apoptosis mainly through suppressing NF-κB activity in tumor tissues. Additionally, DNA microarray data revealed global changes in the gene expression associated with cell proliferation, apoptosis, invasion and metastasis in response to RB treatment. Therefore, our findings suggested that RB exerted its anti-tumor effect by targeting the NF-κB pathway in PCa cells, and this could be a general mechanism for the anti-tumor effect of RB in other types of cancers as well.

## Introduction

Prostate cancer (PCa) is one of the most common malignant tumors in males [1]. It proceeds from a localized, androgen-dependent disease to the invasive and metastatic hormone-refractory prostate cancer (HRPC), without any significant prognostic benefit to conventional antitumor agents [2]. Therefore, novel strategies targeting the molecular basis of PCa progression are urgently required.

The pivotal nuclear factor κB (NF-κB), a well-documented transcriptional factor, is critically important for control of cell proliferation in mammals. In classical pathway, the typical NF-κB dimers (p50/p65) are normally sequestered by binding to IκBα in the cytoplasm. The IκBα subunit is phosphorylated at serine residues 32 and 36 by the IKK, and then degradation through the proteosomal pathway, the p50-p65-IκBα heterotrimer turning into the p50–p65 heterodimer. The nuclear localization signals of NF-κB protein are exposed and its p65 subunit is phosphorylated, leading to nuclear translocation and transcriptional activation potential, and finally inducing the expression of a large number of target genes.[3], [4] Compelling evidence has been demonstrated that aberrant NF-κB regulation is associated with initiation and progression of various types of human cancer, including PCa, by regulating the expression of genes important for many steps of tumorigenesis and progression [2]. For example, the typical NF-κB genes Bcl-2 and survivin, correlated with cell survival; cyclin D1, correlated with proliferation; cyclooxygenase-2 (COX−2), correlated with inflammation; matrix metalloproteinase−9 (MMP−9) and intercellular adhesion molecule (ICAM), correlated with invasion; vascular endothelial growth factor (VEGF) and plasminogen activator urokinase (PLAU), correlate with angiogenesis [3], [4], [5]. It is observed that nuclear localization of NF-κB p65 in primary tumors samples [6], [7], suggesting that constitutive NF-κB activation maybe an early event in PCa development and have prognostic importance for primary tumors. Therefore, intercepting NF-κB signaling might be an attractive antitumor approach [4], [5], [8], [9]. Suppression of NF-κB activity has been shown to repress growth of a variety of cancer cells both *in vitro* and *in vivo*. Furthermore, the anti-apoptotic activity of NF-κB plays a role in the resistance of tumor cells to chemotherapeutic reagents and radiation therapy [8]. In chemoresistant androgen-independent PCa cells, NF-κB is constitutively activated due to the constitutive IκB kinase (IKK) activity [4], [5].

Our previous study have shown that retigeric acid B (RB), a natural pentacyclic triterpenic acid, possesses ability to inhibit cell growth and induce apoptosis in multiple cell lines, including of PCa cells [10]. Down-regulation of the expression of Bcl-2, which is one of the NF-κB-dependent genes, and activation of caspase signaling are observed in RB-treated PC3 cells [10], [11]. Additionally, similar to the structure of RB, other plant-derived triterpenoids including of acetyl-11-keto-β-boswellic acid (AKBA), ursolic acid and betulinic acid, have been reported to interfere with the NF-κB pathway [12], [13], [14]. These finding prompted us to test the hypothesis that RB may exert its anticancer effects in PCa through modulation of NF-κB signaling. In the present study, we demonstrated that RB down-regulated p65 phosphorylation and nuclear translocation, and blocked the constitutive activation of NF-κB signaling in androgen-independent PC3 and DU145 cells and in C56BL/6 homografts mice.

## Results

### RB exhibits inhibitory effect on p65 phosphorylation in carcinoma cells

**Figure 1:**
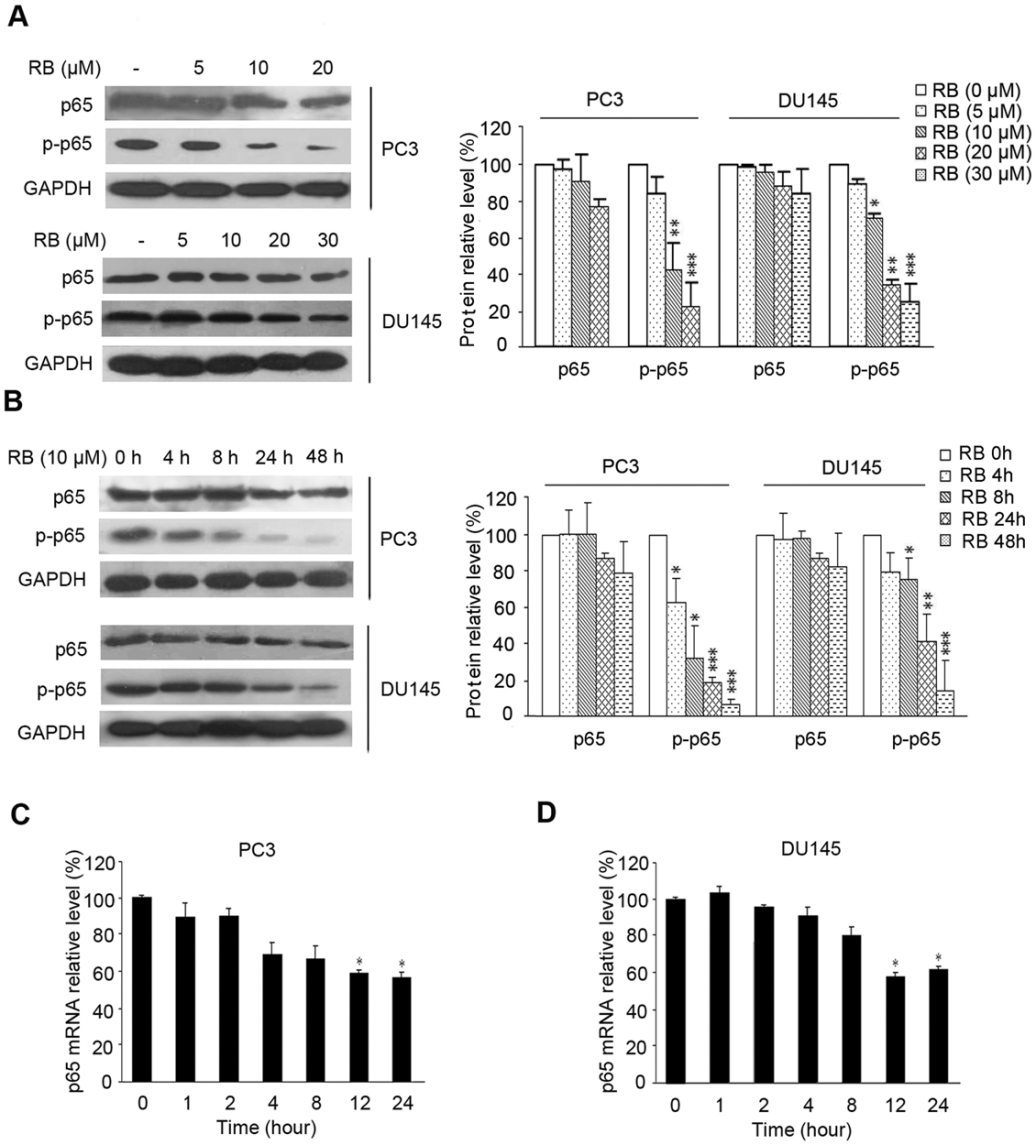
The effect of RB on p65 expression and phosphorylation in PCa cells. (A) and (B) RB slightly inhibited expression of p65 in PC3 and DU145 cells, while significantly for the Ser-536 phosphorylation of p65 (*p–p65*), in both dosage-dependent and time-dependent manner. Lysates from whole cells treated with RB of different concentrations for 24 h (A) or with 10 µM RB for indicated times were used for western blot (B). GAPDH served as the loading control. Protein amount was normalized to the amount of GAPDH, and was quantified by densitometry of X-ray films. Results of one of at least three independent experiments are shown. (C) and (D) RB moderately down-regulated p65 mRNA level in PC3 and DU145 cells as detected by QRT-PCR assay. The procedure was performed as described in *Materials and Methods*. Results shown are representatives of three independent experiments, p<0.05 (*), versus RB-untreated control group respectively.

We initiated the study to test whether RB triggered apoptosis through inhibiting the expression and activation of NF-κB in PC3 and DU145 cells in which NF-κB signaling is constitutively activated and contributed to their resistance to apoptosis due to expression of NF-κB-modulated antiapoptotic proteins. As shown in Figure 1A and 1B, RB showed slight inhibitory effect on the expression of total p65 subunit of NF-κB in PC3 and DU145 cells (1.1∼1.3-fold decrease for the highest dose), whereas significantly down-regulated phosphorylation of p65–Ser536 was both dose- and time-dependent (4∼6-fold decrease for the highest dose or 48 h). Similar to the observations of the protein levels of total p65, quantitative PCR data revealed that the mRNA level of p65 was inhibited by RB treatment in a moderate manner (Figure 1C, 1D). Treatment of PC3 and DU145 cells with 10 μM of RB for 24h resulted in a ∼35% decrease of the mRNA level of p65 compared to the vehicle control (Figure 1C, 1D).

To extend our observation of decreased phosphor-p65 in PCa cells, we detected the levels of total p65 and phosphor-p65 in other cancer cell lines. The results revealed that RB was able to reduce p65 phosphorylation in a panel of carcinoma cell lines including Human liver hepatocellular cells HepG2 cells, human myeloid leukemia cell line K562 cells and adriamycin-resistant K562/AO2 cells in indicated dose, whereas slight effect on human ovarian cancer SKOV3 and human lung adenocarcinoma A549 cells (Figure S1). Together, the data suggested that RB played a profound effect on suppression of p65 phosphorylation, especially in PCa cells.

### RB suppresses the nuclear translocation and activation of NF-κB in PCa cells

**Figure 2:**
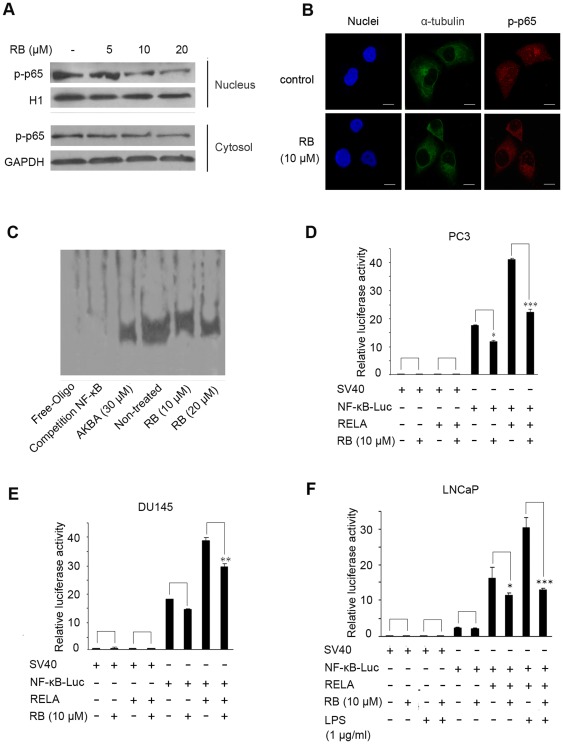
The effects of RB on function of NF-κB *in vitro*. (A) RB dosage-dependently inhibited the nuclear localization of p65 Lysates from cytoplasm and nucleus respectively after treating of PC3 cells with RB for 24 h were used for western blot. GAPDH and H1 respectively served as the loading control. (B) Immunofluorescence analysis of the inhibitory nuclear localization of p65 by RB-treatment for 12 h in PC3 cells. For confocal microscopy, α-tubulin and p-p65 were immunostained, with nuclei stained with DAPI. (Scale bar, 10 μm). (C) Pretreatment of PC3 cells with RB inhibited binding of nuclear extracts to the NF-κB binding site, as detected by electrophoretic mobility shift assay. Lysates from nuclei after treating of PC3 cells with RB for 24 h were used for EMSA. (D), (E) and (F) RB decreased NF-κB activation in PC3 and DU145 cells, and inhibited LPS-induced NF-κB activation in LNCaP cells. The inhibition was more significant when pNF-κB-Luciferase and RELA expression vector co-transfected. Transiently transfected cells were preincubated with RB for 24 h. The luciferase assay was done as described in *Materials and Methods*. In (D)–(F), results are the mean ± S.E. of three independent experiments, each performed in triplicate. p<0.05 (*), p<0.01 (**), p<0.001 (***) versus RB-untreated control group respectively.

We then tested whether RB-mediated suppression of phosphor-p65 led to a decrease in nuclear localization of p65 in PC3 cells, which is necessary for NF-κB to activate target gene transcription. As shown in Figure 2A, RB dramatically reduced the abundance of phosphor-p65 in the nucleus in a dose-dependent manner, and the decreased levels of phosphor-p65, to some extent were observed in cytoplasm as well. The immunofluorescence results in Figure 2B further confirmed that, similar to the western blot assay, the significantly decreased nuclear accumulation of p65 was clearly observed, whereas p65 was diffusely presented throughout the cytosol and the nucleus in untreated cells.

Electrophoretic mobility shift assay (EMSA) was next done to test whether RB affected NF-κB DNA binding ability in PC3 cells. A strong band shift was detected when nuclear extract from untreated control cells (lane 4 in Figure 2C), whereas binding complexes were noticeably reduced in RB-treated cells in dose-dependent manner (Figure 2C, lanes 5 and 6). The specificity of NF-κB binding was confirmed in competition experiments with a 100-fold molar excess of unlabeled NF-κB (Figure 2C, lane 2). AKBA, served as a positive control, decreased the binding activity of NF-κB as well (Figure 2C, lane 3).

In an attempt to determine whether RB correspondingly inhibited NF-κB transcriptional activity, we performed transient transfection assays using a reporter plasmid containing 4 tandem κB binding sites upstream of the luciferase gene. As shown in Figure 2D, RB caused a significant decrease (p<0.05) in NF-κB reporter activity in PC3 cells as compared to the untreated cells. Ectopic expression of p65 (RELA) resulted in a significant up-regulation of NF-κB luciferase activity, which can also be greatly decreased (p<0.001) after treatment with RB (Figure 2D). Similar results were obtained in DU145 cells under the same experimental conditions (Figure 2E).

To investigate that RB was able to suppress inducible NF-κB activity, we performed NF-κB reporter assay in LNCaP cells. Unlike PC3 or DU145 cells, LNCaP cells are known to have low NF-κB activity. As shown in Figure 2F, NF-κB-dependent reporter activity was slightly increased after treatment with NF-κB-inducing factor LPS alone. However, luciferase production was profoundly augmented in cells by overexpression of p65 (RELA). The activity of NF-κB was further dramatically decreased in cells exposed to RB. These data demonstrated that RB suppressed both the inducible (by LPS) and constitutive NF-κB activity in PCa cells, possibly through RB-mediated down-regulation of phosphorylation, nuclear translocation and DNA binding ability of NF-κB.

### RB inhibits IκBα phosphorylation and its degradation

**Figure 3:**
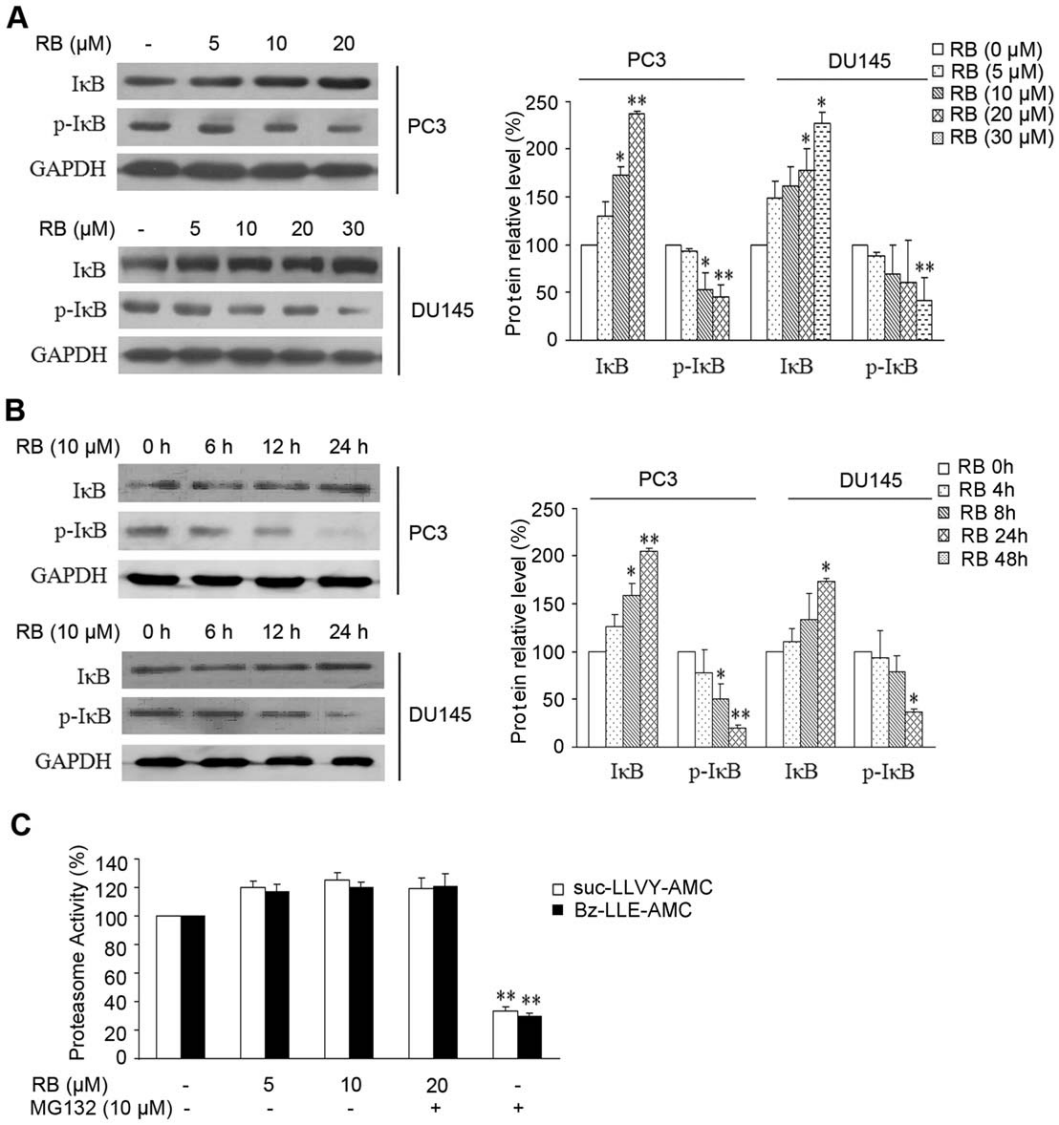
RB inhibits phosphorylation and degradation of IκBα. (A) Western blot analysis of expression of total IκBα and phosphor-IκBα (*p–IκBα,* Ser32/36)p-IκBα. PC3 and DU145 cells were treated with RB of different doses as indicated. (B) Western blot analysis of expression of total IκBα and p-IκBα. PC3 and DU145 cells were treated with RB of different times as indicated. In (A) and (B), equal protein loading was evaluated by GAPDH. Protein amount was normalized to the amount of GAPDH, and was quantified by densitometry of X-ray films. Results of one of at least three independent experiments are shown. (C) The effect of RB on the purified 20S proteasome *in vitro*. MG132 served as positive control. Results are the mean ± SD of three independent experiments, p<0.05 (*), p<0.01 (**), versus untreated control group respectively.

To investigate whether RB-mediated inactivation of NF-κB was ascribed to the inhibition of IκBα degradation, we examined the change of IκBα in response to RB by western blot analysis. As shown in Figure 3A, treatment with increased concentrations of RB resulted in overexpression of total IκBα in both PC3 and DU145 cells. Correspondingly, reduced IκBα phosphorylation was noted after treatment with 10–20 µM of RB in these two cell lines. Time kinetic studies revealed that RB up-regulated the expression of total IκBα, and caused a decrease in the phosphorylation of IκBα from ∼12 h in PC3 cells (Figure 3B). Similar effect was observed in DU145 cells after longer (24 h) treatment with RB than that in PC3 cells (Figure 3B). Proteasome activity assay revealed that RB did not exert inhibitory effect on recombinant 20S proteasome enzyme (Figure 3C), which is required for phosphorylatoin-dependent IκBα degradation. Thereby the data suggested that down-regulated of IκBα degradation and p65 phosphorylation may be due to the effective suppression of IKK activation by RB, which eventually lead to the inhibition of NF-κB activity.

### RB represses the expression of NF-κB-regulated genes, induces apoptosis, and decreases cell migration through the inhibition of NF-κB pathway

**Figure 4:**
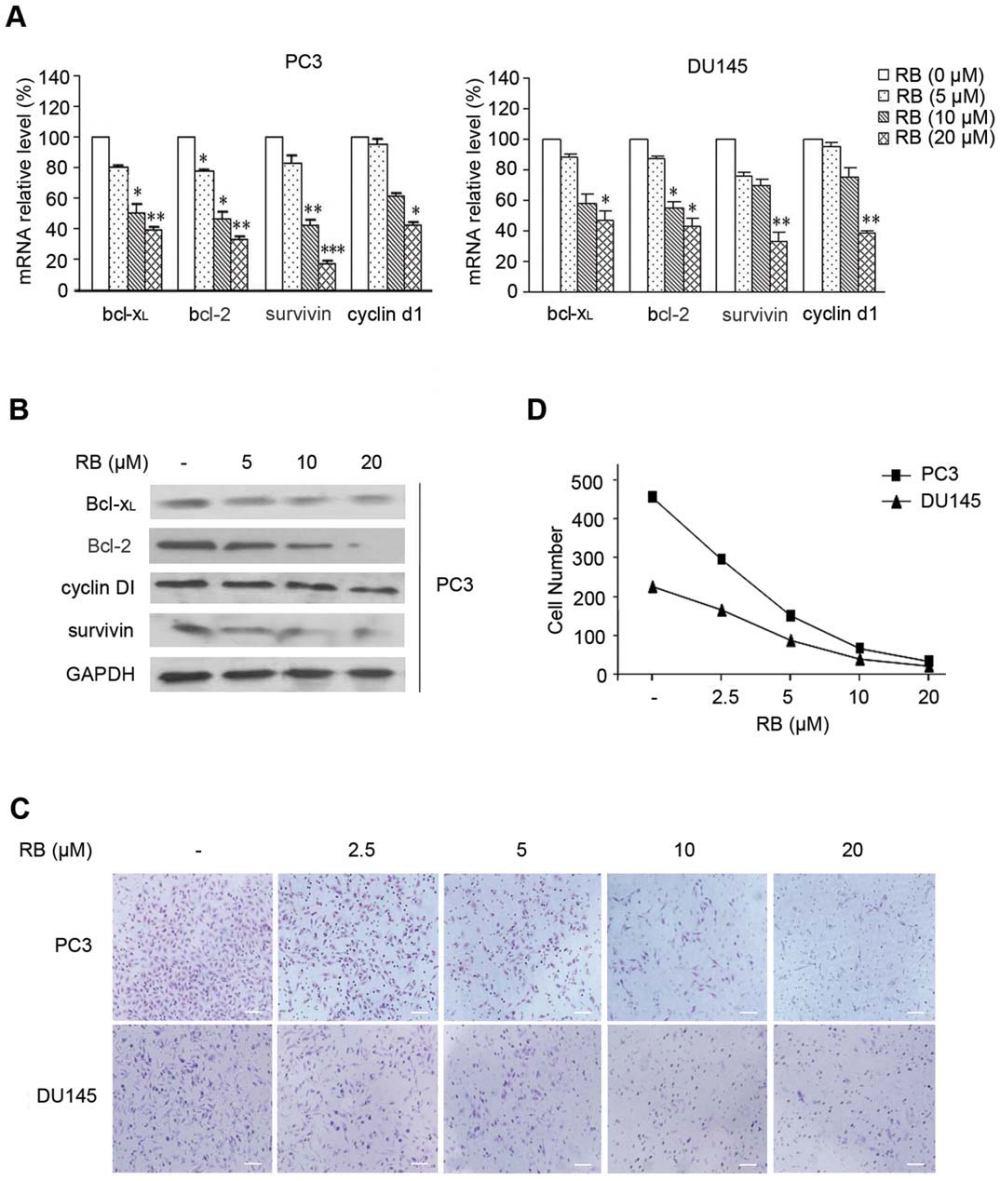
The effects of RB on function of NF-κB-dependent gene expresion and cell invasion *in vitro*. (A) RB dosage-dependently inhibited the mRNA expression of Bcl-x_L_, Bcl-2, survivin, and cyclin D1 as analyzed by QRT-PCR. GAPDH was used for normalization. Results are the mean ± SD of three independent experiments, each performed in triplicate. p<0.05 (*), p<0.01 (**), p<0.001 (***) versus RB-untreated control group respectively. (B) RB dosage-dependently inhibited expression of Bcl-x_L_, Bcl-2, survivin, and cyclin D. The results of western blot analysis of whole cell lysates from PC3 cells treated with different doses of RB were shown; GAPDH was included as a loading control. (C) RB inhibited invasion of PCa cells in a concentration-dependently manner as detected by transwell assay, (scale bar, 100 μm). The procedure was described in *Materials and Methods*. (D) The number of cells that invade through matrigel.

Since NF-κB regulates a variety of genes that are involved in the cell growth, apoptosis, angiogenesis and metastasis of tumor cells, we investigated whether inhibition of NF-κB activity by RB could transcriptionally lead to the modulation of these gene products. Treatment of RB significantly decreased the expression of Bcl-x_L_, Bcl-2, survivin, and cyclin D1 at both mRNA (Figure 4A) and protein levels (Figure 4B) in either PC3 or DU145 cells in a dosage-dependent manner. Survivin, a known antiapoptotic protein, was also markedly reduced in RB-treated cells as shown in Figure 4B.

Inhibition of NF-κB by RB led us to further analyze the impact of RB on cell invasion. As shown in Figure 4C, the invasiveness through matrigel was concentration-dependently decreased in RB-treated PC3 and DU145 cells when compared to vehicle treated cells. As summarized in Figure 4D, RB treatment resulted in significant block of cell migration in a dosage-dependent manner. These results indicated that reduced activation of NF-κB by RB subsequently suppressed NF-κB-dependent antiapoptotic and invasive proteins, leading to the decrease of tumor cell invasion.

To evaluate the changes of global gene expression in PC3 cells after treatment with RB, Affymetrix GeneChip Human Genome U133 Plus 2.0 array, which contained approximately 39,000 characterized human genes, were used to perform the experiment. A total of 855 genes were upregulated greater than two-fold change, and 763 genes downregulated in RB-treated PC3 cells compared to control cells. The results in supplementary data (Table S1) revealed that mRNA levels of NF-κB family and NF-κB-associated genes, in addition to other cell proliferation- and survival-associated genes, and apoptotic regulatory genes presented obviously alteration (> = 1.5-fold). These results are mostly in line with our data described. Many typical genes, such as Bcl-x_L_, cyclin D1, p27, p21, etc. were down-regulated in RB treated sample (Figure S2), therefore, further confirmed RB inhibits NF-κB signaling and its downstream target gene expression.

**Figure 5:**
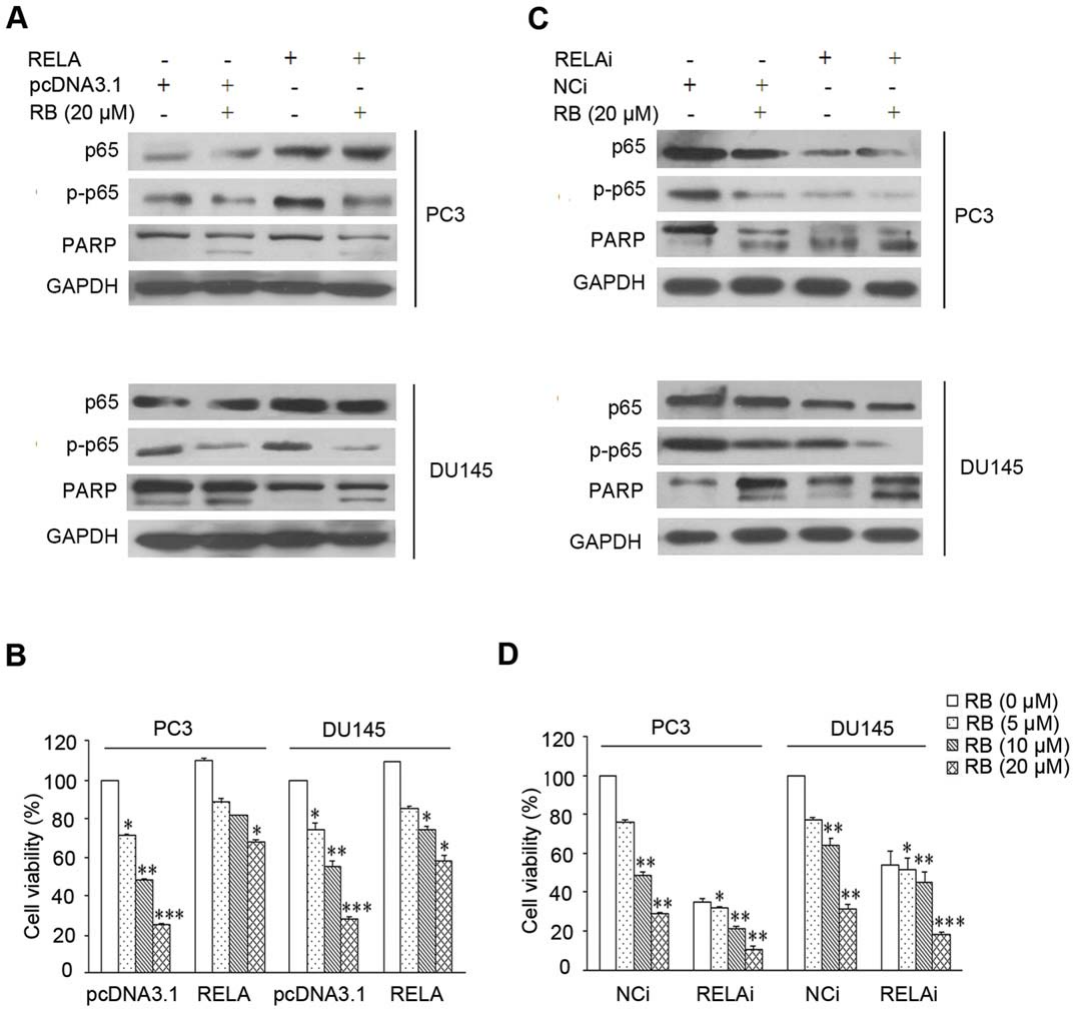
RELA is involved in RB-induced apoptotic cell death. (A) and (B) RELA overexpression by transfection of a RELA expression vector partially abolished cytotoxicity in PC3 and DU145 cells. Cells were transfected with siRNA of RELA (RELAi) or with a negative control siRNA (NCi), using a 50 nmol/L final siRNA concentration. After 48 h transfection for RELA overexpression, the effect of RB on p65, p–p65, and PARP expression in RELA transfected cells was determined by western blot; cell viability was also determined after additional exposure for 24 h to RB. (C) and (D) Silencing of RELA expression by siRNA increased cytotoxicity induced by RB. Similar procedure was performed for the studies on RELA. The details are described in *Material and Methods*. In (A) and (C), the protein levels of p65 and p–p65 were normalized with GAPDH. Results shown are representatives of three independent experiments. In (B) and (D), p<0.05 (*), p<0.01 (**), p<0.001(***) versus RB-untreated control group, respectively.

To determine whether p65 (RELA) plays an important role in RB-inducted apoptosis in PCa cells, we tested the effect of RB on cell viability in cells that are either over-expressed with or ablated for p65 by siRNA. It was observed that p65 expression plasmid indeed enhanced the expression and phosphorylation levels of p65 over the basal levels (Figure 5A), and resulted in a partial rescue of the consequences for RB-induced cell death in both PC3 and DU145 cells (Figure 5B), indicating that p65 conferred cell resistance to apoptosis induced by RB.

In the siRNA experiment, we chose the one of p65-targeting siRNA that exerted the strong activity to deplete p65 expression for evaluation of cell survival (data not shown). As shown in Figure 5C, the expression and phosphorylation of p65 was noticeably abolished in cells transfected with p65-targeting siRNA, as compared to the scramble siRNA. The knockdown of p65 significantly suppressed cell growth (Figure 5D), and RB treatment resulted in much lower level of phosphor-p65 and PARP (116 kDa) in p65-depleted cells and (Figure 5C), leading to the more pronounced inhibition on cell viability. Therefore, these results indicated that NF-κB p65 was essential for RB-induced cell death.

### RB exerts its anti-tumor activity through suppression of p65 phosphorylation in the tumor tissue of mice

**Figure 6:**
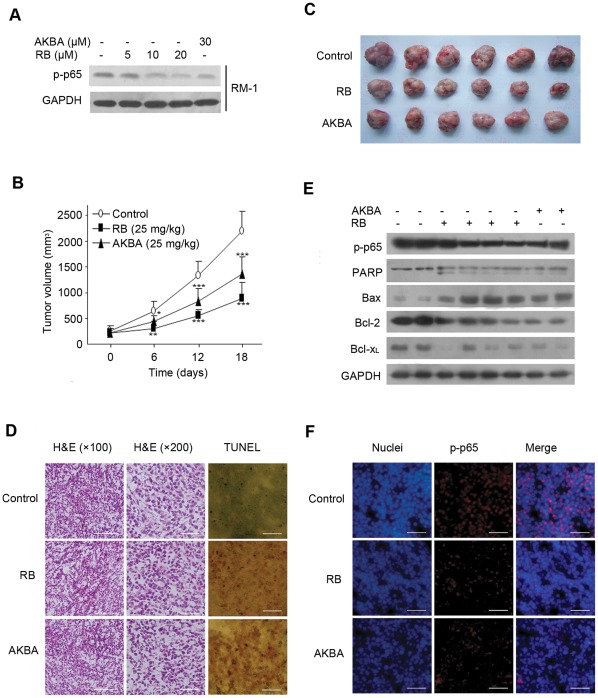
RB inhibits growth of RM-1 homografts in C57BL/6 mice and triggers apoptosis. (A) RB and AKBA concentration-dependently inhibited the Ser-536 phosphorylation of p65 in RM-1 cells. GAPDH served as the loading control. (B) RB (25 mg/kg) reduced the tumor volumes in RM-1-bearing C57BL/6 mice versus the control group, AKBA serving as positive control. 0.2×10^6^ RM-1 cells were s.c. injected into the dorsal region of right side, and after 7 days, the animals were treated intraperitoneally with RB (25 mg/kg), AKBA (25 mg/kg) or medium alone (control) once daily for 18 days, as described in *Materials and Methods*, n = 6 per group; mean ± SD; p<0.05 (*), p<0.01 (**), p<0.001(***) versus medium control group, respectively. (C) Representative tumors from the three groups are shown. (D) H&E and TUNEL-dependent staining of RM-1 tumor tissue sections. (Scale bar, 100 μm). (E) Western blot analysis of p–p65, PARP, Bax, Bcl-2, and Bcl-x_L_ expression in 2 tumor tissue samples treated with medium, 4 samples with RB and 2 samples with AKBA. (F) Immunofluorescence for p–p65 analysis of tumor tissues. (Scale bar, 100 μm).

Having demonstrated the efficacy of RB for blocking NF-κB signaling in PCa cells in culture, we next investigated its potential antitumor effect in mice. In particular, we performed studies to investigate the expression and activation of p65 in tumor tissues of mice. Firstly we examined the cytotoxic activity of RB and AKBA. RB and AKBA remarkably reduced RM-1 cell growth (data not shown). More importantly, RB and AKBA significantly suppressed phosphorylation of p65 in RM-1 cells at desired concentrations (Figure 6A). Thus, this cell line is equivalent to its human counterparts and was used for establishment of PCa homografts in male C57BL/6 mice. After planting RM-1 cells subcutaneously into C57BL/6 mice, we treated mice by daily intraperitoneal injections with 25 mg/kg of RB started 7 days after cell inoculation and continued for another 18 days. Group of treatment with 25 mg/kg of AKBA served as the positive control. Compared to receiving vehicle control group, RB- or AKBA-treatment significantly reduced the average tumor volume after 6th, 12th and 18^th^ days of treatment, RB group displayed a more powerful potential (Figure 6B and 6C). The results in Table 1 revealed that, tumor weights were also decreased significantly in the RB and AKBA groups in comparison with the vehicle control group (p<0.001). The inhibition rates of tumor weight of RB and AKBA groups were 49.60% and 30.01%, respectively. TUNEL staining demonstrated that, in contrast to the tumor tissues receiving vehicle, increasing numbers of TUNEL positive stained apoptotic cells were seen in the tumor tissues from RB or AKBA treated mice (Figure 6D, right panel). These apoptotic cells were recognized in the whole section of the tumor issues treated with chemicals. Histological H&E staining (Figure 6D) showed the morphologic changes in the tumor tissues. In contrast to control group, RB or AKBA treatment caused an increase in the fraction of apoptotic cells with shrinkage cytoskeleton and condensed nucleus, suggesting that tumor tissues responded to RB or AKBA with increased rates of apoptosis.

**Table 1:** Effect of RB on tumor growth (n = 6, mean ± S.D.)

	Group		
	Control	RB (25 mg/kg)	AKBA (25 mg/kg)
Tumor weight (g)	2.38±0.21	1.19±0.29***	1.66±0.26***
Inhibition rate (%)		49.60***	30.01***

*** P<0.001 compared with Control group.

To gain insights in the mechanistic basis for RB's antitumor activity, we investigated the expression status of various proteins associated with apoptosis and particularly activation of NF-κB in the target tissues. As shown in Figure 6E, RB produced a profound inhibition of PARP, Bcl-2, and Bcl-xL expressions in tumor tissues from treated mouse groups, whereas caused a striking increase in the expression of Bax, consistent with the results in culture cells. Notably, delivered RB in mice suppressed activation of NF-κB, as indicated by the reduction of phosphor-p65 expression in tumor tissues when compared to the vehicle control groups. Immunofluorescence analysis of the tumor sections for phospho-p65 revealed that, similar to the effect of AKBA, RB notably inhibited phosphorylation and localization of p65 *in situ* (Figure 6F). Together, the data demonstrated that RB exerted antitumor activity against PCa homografts in C57BL/6 mice, by inactivation of NF-κB signaling and induction of apoptosis.

## Discussion

RB, a member of natural pentacyclic trieroenic acids, has been recently gained our attention for its potential antitumor activity in PCa [10].

In this study, we found that RB was a potent inhibitor of NF-κB in PCa cells. Firstly, RB inhibited phosphorylation of p65 on Ser-536 *in vitro* and *in vivo*. Secondly, RB inhibited IκBα phosphorylation and degradation, eventually leading to the inhibition of nuclear translocation and DNA binding activity of p65. The inhibitory effect on NF-κB activity by RB was also reflected by the reduced expressions of NF-κB-dependent genes such as *bcl-2, bcl-x_L_, cyclin d1,* and *survivin*, and the final consequence of the inhibition of NF-κB in PCa cells. Overexpression and knockdown experiments supported the observations that the reduction of p65 by RB was crucial in its apoptotic modulation. In addition to PCa cells, suppression of NF-κB activation by RB was observed in other different cancer cell lines, including myeloid leukemia cells (K562, AO2), and liver hepatocellular cells (HepG2), indicating that inactivation of NF-κB by RB is a general mechanism for its antitumor activity.

As known, the mechanism of NF-κB activation in cells on stimulation is primarily dependent on the phosphorylation and ubiquitination of the inhibitory proteins IκBs and subsequent subjected for degradation by the 26S proteasomes. We found that RB had no effect on the activity of 20S proteasome, suggesting that inhibition of IκB degradation and inactivation of NF-κB p65 by RB was mainly due to a step upstream of IκBα phosphorylation. The classic regulation pattern is inhibition of IKK activity, which in turn leading to the reduction of phosphor-IκBα. Whether IKK is direct target of RB and inactivation of IKK by RB is essential for its inhibitory effects on IκB/ NF-κB remains to be elucidated in the future work. In addition to classical NF-κB, RB didn't alter the expression of p50 and p52 in PCa cells (data not shown), suggesting that the nonclassical NF-κB pathway is not essential in RB-mediated cell death.

As expected, in this study we found the pattern of activity modulation of RB was similar to that of other natural pentacyclic triterpenic acids. AKBA, ursolic acid and betulinic acid directly or indirectly interact with IKK and inhibit NF-κB signaling [12], [13], [14]. This biological characteristic is observed in a variety of different cell lines, including human PCa (PC3 and DU145), human leukemia (Jurkat cells), human embryonic kidney (293 cells), human myelogenous leukemia (KBM-5), human non-small cell lung carcinoma (H1299), and human histiocytic lymphoma (U937), colon cancer (HCT116 and Caco2) and pancreatic cancer (PANC-28) [12], [13], [14], [15], [16], [17]; these findings suggest that natural pentacyclic triterpenic acids can be broad inhibitors of NF-κB activation.

However, suppression of some gene products by pentacyclic triterpenic acids seems to be selective Our data showed that VEGF and COX−2 expressions remained unchanged in PC3 cells exposed to RB (data not shown). Other groups' studies have reported that AKBA suppresses VEGF expression in plasmacytoma U266 cells [18], and inhibits COX−2 mRNA expression induced by TNF in KBM-5 cells [19]. In accordance with AKBA, ursolic acid and betulinic acid also trigger reduction of VEGF and COX−2 level in other cell lines, including human leukemia cell line Jurkat, human epithelial cell line HCT116, lung cancer cell lines A549, H3255 and Calu-6, human PCa cell line PC3, human colon cancer cell lines RKO and SW480. [13], [14], [20], [21]. The effects of plant-derived pentacyclic triterpenic acids on NF-κB signaling pathway may be varied due to the different substituted groups in pentacyclic triterpenes, and/or cell type-dependent.

Collectively, our findings suggest that RB promotes apoptosis through suppression of NF-κB activation and NF-κB-dependent antiapoptotic gene expression in PCa cells and animal models, and NF-κB signaling pathway represents an important molecular target for anticancer activity of RB.

## Materials and Methods

### Chemicals

Retigeric acid B (RB) was isolated from lichen *L. kurokawae*, and its purity and structure determination was described previously [22]. Acetyl-11-keto-β-boswellic acid (AKBA) was isolated and purified by reverse phase high performance liquid chromatography as described previously [22]. The compounds were dissolved in dimethyl sulfoxide (DMSO) at 10 mM as stock solutions stored at −20°C and diluted according to experimental requirements when used. For the application of RB in the homograft models we developed a solvent system consisting of physiological saline/DMSO/ethanol/tween 80 (75∶10∶10∶5, v/v).

### Cell Culture and Homografts

Human PCa LNCaP (The American Type Culture Collection, Rockville, MD), PC3, DU145 cells, and murine PCa RM-1 cells (The Cell Bank of Chinese Academy of Sciences, Shanghai) were cultured in RPMI 1640 medium (HyClone) supplemented with 10% FBS (HyClone). Human lung adenocarcinoma A549 cells were cultured in Ham's /F-12 (HyClone) supplemented with 10% FBS (HyClone). Human liver hepatocellular cells HepG2, human ovarian cancer cells SKOV3, human myeloid leukemia cell line K562 and adriamycin-resistant K562/AO2, were cultured in RPMI 1640 medium (HyClone) supplemented with 10% FBS (HyClone).

RM-1 cells from C57BL mouse prostate tumors are androgen-independent and can be transplanted into the syngeneic mice to reconstitute the homotransplantation, which is suitable model to simulate human PCa. [23], [24] For homotransplantation male C57BL/6 mice of aged 7 weeks (Vital River Laboratories, Beijing) were inoculated subcutaneously in the backs with 0.2×10^6^ RM-1 cells in 0.2 ml of physiological saline [23]. After 1 week, treatment commenced by single daily intraperitoneal injections of RB or AKBA at 25 mg/kg or equivalent amounts of solvent for additional 18 days [12]. The tumor size was measured by 6 days with a caliper; the tumor volume was calculated according to the formula 0.5×L ×W^2^ (L = length, W  =  width). At the end of the experiment, all mice were euthanized by air-ether anesthesia and their tumors resected. The tumors were prepared frozen tissue slices (5 µm thick). Proteins extracted from each tumor tissue were used for western blot. All animal experiments were approved by the Ethics Committee of Shandong University School of Medicine (Permit Number: 2010037) and conducted accordingly.

### MTT Assay

3-(4,5-dimethylthiazol-2-yl)-2,5-diphenyl-2H-tetrazolium bromide (MTT, Sigma) colorimetric assay was used to quantitate PC3, DU145 and RM-1 cells proliferation and cytotoxicity in the presence of RB. Cells (1×10^4^ per well) were seeded into 96-well plates. After 24 hours incubation, the cells were treated with vehicle, or desired concentrations of RB for further 24 hours. After removing the medium, incubate cells with 10 μl MTT for 4 hours. The cell growth response to the chemicals was detected by measuring the light absorbance at 570 nm on a plate reader (Bio-rad, USA).

### Western Blot Analysis

After treatment with RB at desired concentrations for 24 hours or with 10 μM RB for desired times, whole cell lysates was obtained according to the method described previously [25]. The procedures for western blot were described previously [26]. Blots were incubated with primary antibodies against p65, survivin (Santa Cruz Biotechnology), phosphor-p65 (Ser536), phosphor-IκBα (Ser32/36), IκBα, Bcl-x_L_, Bcl-2, Bax, total and cleaved PARP (Cell Signaling Technology), cyclin D1 (NeoMarkers). Membranes were stripped and reprobed with glyceraldehyde-3-phosphate dehydrogenase (GAPDH) (Santa Cruz Biotechnology) as a protein loading control. H1 (Bioworld) served as the loading control for nucleoprotein.

### Quantitative RT-PCR Analysis

Quantitative real-time PCR (QRT-PCR) assay was carried out to analyze the mRNA level of p65 in PC3 and DU145 cells treated with 10 μM RB for desired times, and Bcl-x_L_, Bcl-2, survivin, cyclin D1, PCNA, cyclin B1, p27, p21, H2AX, DDIT4, EGF, ATM and GAPDH with RB-treatment for 24 hours. Total RNAs of PC3 and DU145 cells were extracted by Trizol (TaKaRa). cDNA was synthesized through reverse transcription using ReverTra Ace® qPCR RT Kit (TOYOBO). QRT-PCR was performed using the Eppendorf QRT-PCR System. Primers for p65, Bcl-x_L_, Bcl-2, survivin, cyclin D1, PCNA, cyclin B1, p27, p21, H2AX, DDIT4, EGF, ATM and GAPDH were 5′-GGGAAGGAACGCTGTCAGAG-3′ (forward) and 5′-TACCTCAGGGTACTCCATCA-3′ (reverse); 5′-GGTCGCATTGTGGCCTTCTT-3′ (forward) and 5′-CTCTCGGCTGCTGCATTGTT-3′ (reverse); 5′-TGTTGGCCGGATCACCAT-3′ (forward) and 5′- TCCCCAATGATCAGGTCCTTT-3′ (reverse); 5′-AGGACCACCGCATCTCTACAT-3′ (forward) and 5′-AAGTCTGGCTCGTTCTCAGTG-3′ (reverse); 5′-CAAACAGATCATCCGCAAACA-3′ (forward) and 5′-GCAGTCTGGGTCACACTTG-3′ (reverse); 5′-TGGTCACCAGGGCTGCTT-3′ (forward) and 5′-AGCTTCCCGTTCTCAGCCTT-3′ (reverse); 5′-ACACTAAGGGCCGAAGATAACG-3′ (forward) and 5′-CGGCATATACGTGCAAATTCAC-3′ (reverse); 5′-ATAAGGCGAAGATCAACATGGC-3′ (forward) and 5′-TTTGTTACCAATGTCCCCAAGAG-3′ (reverse); 5′-TAATTGGGGCTCCGGCTAACT-3′ (forward) and 5′-TTGCAGGTCGCTTCCTTATTC-3′ (reverse); 5′-CCTGTCACTGTCTTGTACCCT-3′ (forward) and 5′-GCGTTTGGAGTGGTAGAAATCT-3′ (reverse); 5′-TTGGTAACAGGCACATCTTCCT-3′ (forward) and 5′-TTCCGCAAAACGACTCTTG-3′ (reverse); 5′-AGCGGCAGGACGCACTTGTC-3′ (forward) and 5′-GGCCGATCTGGGGTGGGAGT-3′ (reverse); 5′-CAAACACACTGGAAAGGACATGG-3′ (forward) and 5′-ATCTTCTGCCTTGGGTTGTGC-3′ (reverse); 5′-TGATGCTTTCTGGCTGGATTT-3′ (forward) and 5′-GCAGCACAAGACTGAGCTACC-3′ (reverse), respectively. GAPDH gene was co-amplified to serve as an internal control.

### Electrophoretic Mobility Shift Assay (EMSA) and Nuclear Translocation of Transcription Factor p65

The oligonucleotides used for an NF-κB binding site (Promega), were 5′-AGTTGAGGGGACTTTCCCAGGC-3′, labeled with digoxigenin at 3′ end by the terminal transferase method. Nuclear extracts (10 μg/sample) from PC3 cells with or without pretreatment with RB or AKBA for 24 hours were subjected to EMSA following the protocol provided by Roche [22]. For competition studies, nuclear extracts (Nuclear and Cytoplasmic Protein Extraction Kit, Beyotime) were incubated on ice for 30 minutes with a 100-fold excess of unlabeled specific NF-κB oligonucleotides. AKBA (30 uM) served as a positive control. In experiments of nucleic localization of phosphor-p65 in PC3 cells, cytosol and nuclear lysates extracted using Nuclear and Cytoplasmic Protein Extraction Kit (Beyotime) were used for western blot. GAPDH and H1, respectively served as the loading control.

### Transient Transfection Assay

PC3, DU145 and LNCaP cells were seeded in 24-well plates and grew under the conditions described above. The vectors pNF-κB-Luciferase (NF-κB promoter, containing 4 tandem κB binding sites upstream of the luciferase gene, 0.5 μg/well), or RELA expression vector (0.15 μg/well) were transfected into cells using Lipofectamine2000 (Invitrogen Life Technologies). The SV40 vector (0.8 μg/well) was used as a control. The phRL-TK vector (0.05 μg/well, Renilla luciferase, Promega) was co-transfected to normalize transfection efficiency. After 24 hours the cells were exposed to RB for an additional 24 hours. Cell lysates were prepared for luciferase assays (Dual-luciferase reporter assay system, Promega). In experiments with lipopolysaccharide (LPS, Sigma), 1 µg/ml of LPS was used to treat cells for 30 minutes after the addition of the RB. In experiments to detect the cytotoxicity and apoptosis parameters of RB after transfecting RELA, MTT assay was performed after pretreating with RB for 24 hours. Cells transfecting pcDNA3.1 (0.8 μg/well) served as the control.

### Transwell Invasion Assays

Transwell invasion assays were performed with Growth Factor Reduced Matrigel Invasion Chambers (BD Biosciences) according to the manufacturer's protocol. Briefly, PC3 or DU145 cells pretreated with RB of different concentrations for 24 hours were dissociated from the plates and 0.5×10^6^ cells were resuspended in RPMI-1640 containing 1% bovine serum albumin into the upper chamber in which matrigel matrix (BD Biosciences) was added. RPMI-1640 containing 10% fetal bovine serum as a chemoattractant was added to each bottom well. Cells were allowed to invade for 48 hours at 37°C, then non-invaders were removed. Invading cells were fixed and stained with the Gimsa stain solution. Cells were counted, and the total number of cells per chamber was used for calculating the number of invading cells.

### Gene Silencing with Small Interfering RNA

Gene silencing was achieved by small interfering RNA (siRNA) of RELA transfection. Sense sequences of three RELA siRNAs (synthesized from Invitrogen) are 5′ -UUCACUCGGCAGAUCUUGAGCUCGG-3′, 5′-AAACUCAUCAUAGUUGAUGGUGCUC-3′, 5′-UUUACGUUUCUCCUCAAUCCGGUGA-3′, respectively. Negative control siRNA oligonucleotides (synthesized from Invitrogen) target the sequence 5′-AATTCTCCGAACGTGTCACGT-3′. After transfecting the three siRNAs (50 nmol/L) into PC3 cells respectively, and incubating for 48 hours, we evaluated their effects on RELA silencing via western blot analysis and obtained the one with most remarkable gene silencing effect. Then we treated PC3 and DU145 cells with RB, as described previously, 48 hours after transfected the cells using the RELA siRNA and the negative control siRNA with Lipofectamine2000 (Invitrogen) according to the manufacturer's instruction. Effects of RB on the conditioned cells were evaluated by western blot analysis and cell survival assay.

### Proteasome activity assay

Proteasome activities were measured via the direct addition of RB to purified 20S proteasome *in vitro*. The substrates N-Succinyl-Leu-Leu-Val-Tyr-7-amino-4-methylcoumarin (suc-LLVY-AMC, ML-P802) and Benzyloxycarbonyl-L-leucyl-leucyl-glutamyl-methylcoumarylamide (Bz-LLE-AMC, ZW9345) were incubated at 37°C for 40 minutes with pure 20S proteasome (human erthrocyte, PW8720, ENZO Life Sciences), untreated or pretreated 5 µM, 10 µM RB or 10 µM MG132 (#474790, Calbiochem) in assay buffer. Fluorescene was determined by Mithras LB-940 (Berthold Technologies, Germany), with an excitation of 355 nm and emission of 460 nm.

### Immunofluorescence, H&E Staining and TUNEL Assay

The frozen sections obtained from mouse tumor tissues were fixed in 4% paraformaldehyde, and processed for H&E staining [27] or immunofluorescence for phosphor-p65 [28], nuclei marked with DAPI (Sigma-Aldrich) staining. For the *in situ* detection of apoptotic cells in tumor tissue sections, the terminal deoxynucleotidyl transferase-mediated dUTP nickend labeling (TUNEL) method was used (DNA Fragmentation Detection Kit, Calbiochem). The sections were counterstained with Methyl green. The images were digitally recorded at a magnification of 100× or 200× with an inverted microscope (Nikon).

For the immunofluorescence analysis of nucleic localization of phosphor-p65 in PC3 cells, the confocal laser-scanning microscopy (Carl Zeiss, LSM780) was used. The cells were treated with 10 μM RB for 12 h. α-tubulin (Cell Signaling Technology) was immunostained as a marker for the cytosol, whereas nuclei were stained with DAPI; antibody against phosphor-p65 (Ser536, Cell Signaling Technology) was used for the experiment.

### Microarray Analysis

For this experiment, we used Affymetrix GeneChip® 3′ expression arrays [29]. Total RNA was isolated after treating PC3 cells with 10 μM RB or DMSO (control) for 24 hours. The procedure followed the protocols developed by Affymetrix, GeneChip 3′ IVT Express Kit. Multi-array analysis (RMA) [29] was performed using Affymetrix Expression Console software, and Partek GS 6.5 software provided the results of data contrast analysis.

### Statistical Analysis

The data are presented as the mean ± S.D. of at least three independent experiments. The statistical significance of mean difference between the control and treated groups was determined by a paired t-test. *P*<0.05 was considered statistically significant.

## Supporting Information

Figure S1 **RB blocks NF-κB activation in a variety of cancer cell types, to a different degree.** We tested levels of total p65 and phosphor-p65 in Human lung adenocarcinoma cells A549, human liver hepatocellular cells HepG2, human ovarian cancer cells SKOV3, human myeloid leukemia cell line K562 and adriamycin-resistant K562/AO2, with indicated dose of RB for 24 h. GAPDH served as the loading control.
(TIF)

Figure S2 **The validation of DNA microarray data by QRT-PCR assay.** We detected the 9 mRNA expression alteration involved in PCNA, cyclin B1, p27, p21, H2AX, DDIT4, EGF, ATM and GAPDH in PC3 cells with RB-treatment for 24 h versus medium-treatment respectively, across the two different methods.
(TIF)

Table S1 **NF-κB family and NF-κB-associated genes, other cell proliferation- and survival-associated genes expression alteration p (> = 1.5-fold) in PC3 cells treated with 10 µM RB for 24 h with the DNA microarray analysis.**
(DOC)
