# Supplementary figures and images for: Retigeric Acid B Exhibits Antitumor Activity through Suppression of Nuclear Factor-κB Signaling in Prostate Cancer Cells in Vitro and in Vivo
Source: PLoS One. 2012 May 29;7(5):e38000. doi: 10.1371/journal.pone.0038000 (PMC3362538; doi:10.1371/journal.pone.0038000)

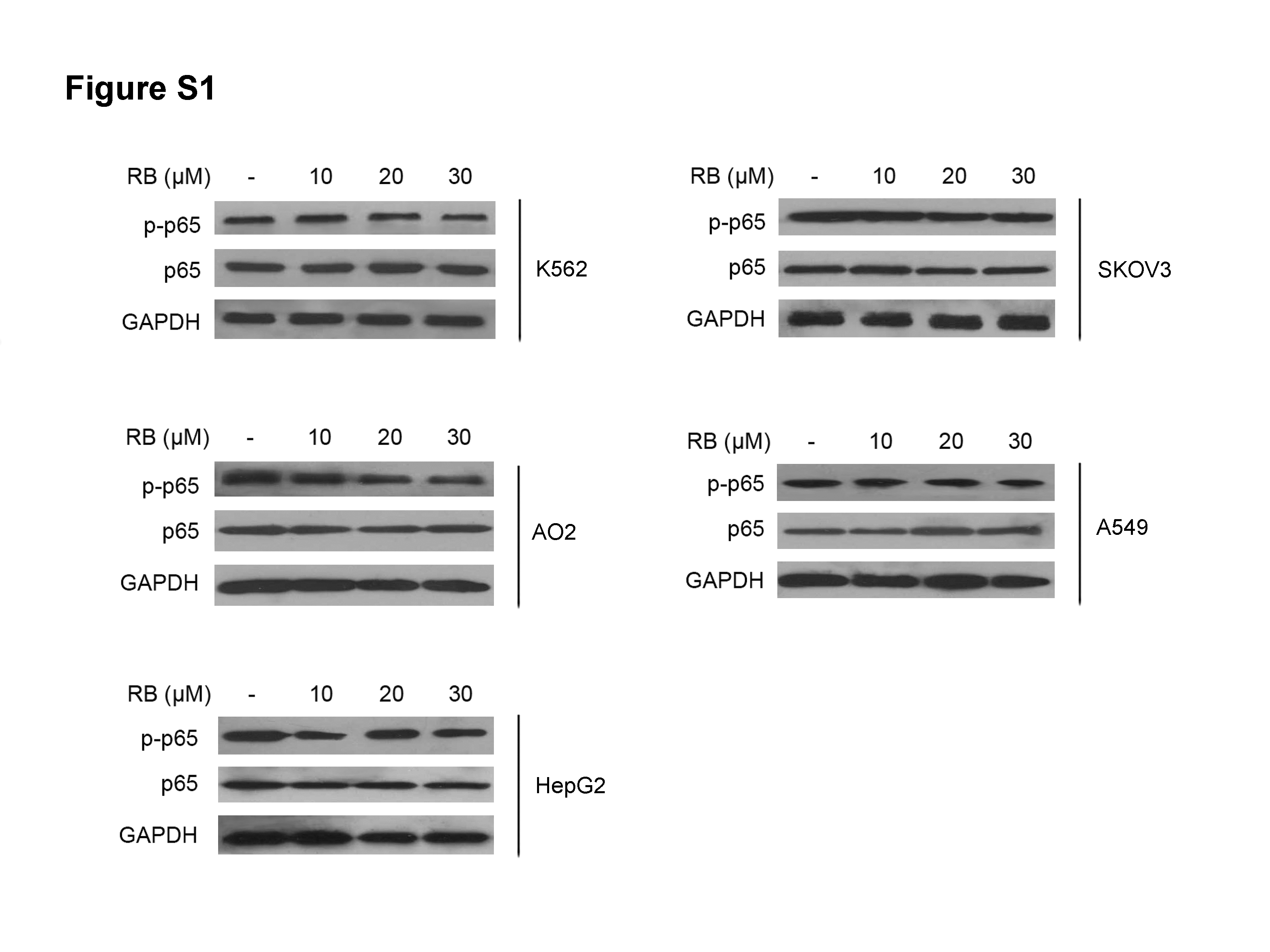

Supplement: Figure S1 — RB blocks NF-κB activation in a variety of cancer cell types, to a different degree. We tested levels of total p65 and phosphor-p65 in Human lung adenocarcinoma cells A549, human liver hepatocellular cells HepG2, human ovarian cancer cells SKOV3, human myeloid leukemia cell line K562 and adriamycin-resistant K562/AO2, with indicated dose of RB for 24 h. GAPDH served as the loading control. (TIF) [file pone.0038000.s001.tif]

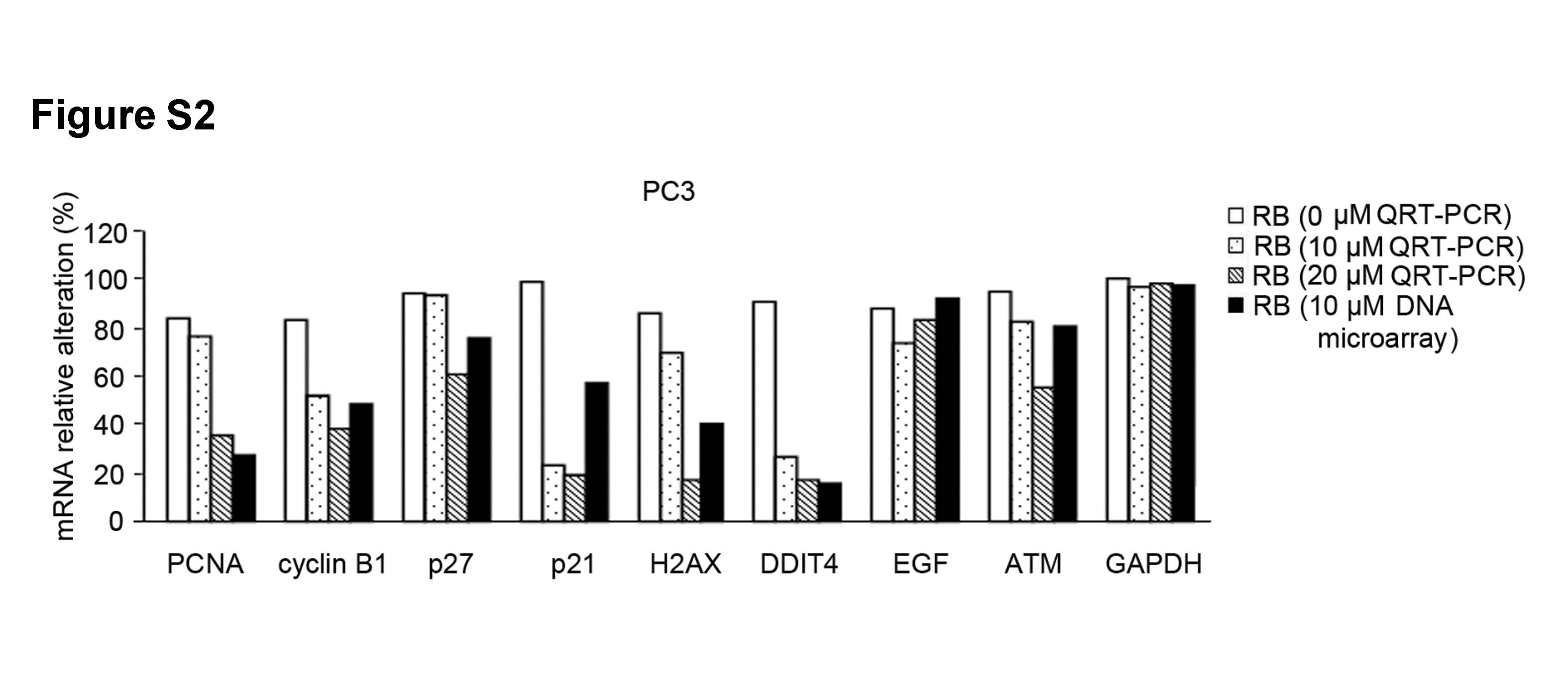

Supplement: Figure S2 — The validation of DNA microarray data by QRT-PCR assay. We detected the 9 mRNA expression alteration involved in PCNA, cyclin B1, p27, p21, H2AX, DDIT4, EGF, ATM and GAPDH in PC3 cells with RB-treatment for 24 h versus medium-treatment respectively, across the two different methods. (TIF) [file pone.0038000.s002.tif]
