# Supplementary material for: Retigeric Acid B Exhibits Antitumor Activity through Suppression of Nuclear Factor-κB Signaling in Prostate Cancer Cells in Vitro and in Vivo
Source: PLoS One. 2012 May 29;7(5):e38000. doi: 10.1371/journal.pone.0038000 (PMC3362538; doi:10.1371/journal.pone.0038000)
Supplement: Table S1 — NF-κB family and NF-κB-associated genes, other cell proliferation- and survival-associated genes expression alteration p (> = 1.5-fold) in PC3 cells treated with 10 µM RB for 24 h with the DNA microarray analysis. (DOC) [file pone.0038000.s003.doc]

Table S1

Gene expression alteration in PC3 cells

treated with 10 µM RB for 24 h (>=1.5-fold)

| Genes | Description | PC3/RB  (Log ratio) |
| --- | --- | --- |
| *NF-κB related* | | |
| NM_001145138 RELA | v-rel reticuloendotheliosis viral oncogene homolog A (avian) | -2.11 |
| NM_003998 NFKB1 | nuclear factor of kappa light polypeptide gene enhancer in B-cells 1 | -3.16 |
| NM_001077493 NFKB2 | nuclear factor of kappa light polypeptide gene enhancer in B-cells 2 (p49/p100) | -1.79 |
| NM_006509 RELB | v-rel reticuloendotheliosis viral oncogene homolog B | -3.25 |
| NM_002908 REL | v-rel reticuloendotheliosis viral oncogene homolog (avian) | -1.09 |
| *Cell cycle and Proliferation* |  |  |
| NM_031966 CCNB1 | cyclin B1 | -2.06 |
| NM_004701 CCNB2 | cyclin B2 | -1.85 |
| NM_053056 CCND1 | cyclin D1 | -2.32 |
| NM_00113601 CCND3 | cyclin D3 | -1.73 |
| NM_001761 CCNF | cyclin F | -2.32 |
| NM_004354 CCNG2 | cyclin G2 | 1.88 |
| NM_001134375 CCNJ | cyclin J | -2.10 |
| NM_001798 CDK2 | cyclin-dependent kinase 2 | -2.52 |
| NM_001145306 CDK6 | cyclin-dependent kinase 6 | -1.94 |
| NM_001260 CDK8 | cyclin-dependent kinase 8 | -1.72 |
| NM_001130829 CDC2 | cell division cycle 2, G1 to S and G2 to M | -3.68 |
| NM_000389 CDKN1A | cyclin-dependent kinase inhibitor 1A (p21, Cip1) | 1.75 |
| NM_000076 CDKN1C | cyclin-dependent kinase inhibitor 1C (p57, Kip2) | 1.80 |
| NM_002592 PCNA | proliferating cell nuclear antigen | -3.71 |
| *Invasion and Metastasis* |  |  |
| NM_001025366 VEGFA | vascular endothelial growth factor A | 2.45 |
| NM_005429 VEGFC | vascular endothelial growth factor C | -1.74 |
| NM_001145031 PLAU | plasminogen activator, urokinase | -7.38 |
| NM_001005376 PLAUR | plasminogen activator, urokinase receptor | -3.09 |
| *Adhesion* |  |  |
| NM_000610 CD44 | CD44 molecule (Indian blood group) | -2.32 |
| NM_002203 ITGA2 | integrin, alpha 2 (CD49B, alpha 2 subunit of VLA-2 receptor) | -1.91 |
| NM_002205 ITGA5 | integrin, alpha 5 (fibronectin receptor, alpha polypeptide) | -1.63 |
| NM_000210 ITGA6 | integrin, alpha 6 | -3.23 |
| NM_000201 ICAM1 | intercellular adhesion molecule 1 | -6.80 |
| *Apoptosis related* | | |
| NM_001040668 BCL2L1 | BCL2-like 12 (proline rich) | -1.80 |
| NM_003921 BCL10 | B-cell CLL/lymphoma 10 | -1.86 |
| NM_182557 BCL9L | B-cell CLL/lymphoma 9-like | -1.93 |
| NM_001166 BIRC2 | baculoviral IAP repeat-containing 2 | -1.60 |
| NM_001165 BIRC3 | baculoviral IAP repeat-containing 3 | -23.54 |
| NM_001012270 BIRC5 | baculoviral IAP repeat-containing 5 | -2.14 |
| NM_001127183 CFLAR | CASP8 and FADD-like apoptosis regulator | -2.11 |
| NM_005658 TRAF1 | TNF receptor-associated factor 1 | -7.95 |
| NM_021138 TRAF2 | TNF receptor-associated factor 2 | -1.78 |
| NM_003300 TRAF3 | TNF receptor-associated factor 3 | -1.90 |
| NM_004295 TRAF4 | TNF receptor-associated factor 4 | 1.66 |
| NM_006290 TNFAIP3 | tumor necrosis factor, alpha-induced protein 3 | -15.96 |
| NM_000660 TGFB1 | transforming growth factor, beta 1 | -2.01 |
| NM_004324 BAX | BCL2-associated X protein | 1.86 |
| NM_001042618 PARP2 | poly (ADP-ribose) polymerase 2 | -1.57 |
| NM_006437 PARP4 | poly (ADP-ribose) polymerase family, member 4 | -1.50 |
| NM_024615 PARP8 | Poly (ADP-ribose) polymerase family, member 8 | -1.52 |
| NM_017554 PARP14 | poly (ADP-ribose) polymerase family, member 14 | -1.81 |
| *Prostate Cancer Markers* |  |  |
| NM_001030047 KLK3 | kallikrein-related peptidase 3 | -1.50 |
| *Others* |  |  |
| NM_002046 GAPDH | glyceraldehyde-3-phosphate dehydrogenase | NC |
| NM_005225 E2F1 | E2F transcription factor 1 | -2.53 |
| NM_004091 E2F2 | E2F transcription factor 2 | -2.29 |
| NM_024680 E2F8 | E2F transcription factor 8 | -5.91 |
| NM_002105 H2AFX | H2A histone family, member X | -2.47959 |
| NM_019058 DDIT4 | DNA-damage-inducible transcript 4 | 6.4363 |
| NM_003109 SP1 | Sp1 transcription factor | -1.66 |
| NM_007358 MTF2 | metal response element binding transcription factor 2 | -2.31 |
| NM_001143820 ETS1 | v-ets erythroblastosis virus E26 oncogene homolog 1 (avian) | -1.80 |

NC, negative control.
